# Angiotensin-Converting Enzyme (ACE) Insertion/Deletion (I/D) Polymorphism and Migraine Susceptibility and Phenotypes: A Clinical-Genetic Study in a United Arab Emirates (UAE) Cohort

**DOI:** 10.3390/jcm15145748

**Published:** 2026-07-22

**Authors:** Eslam ElNebrisi, Mohamed Elshafei, Sarah Safwat, Lamia Ibrahim, Mariam A. L. Younan, Shyam Babu Chandran, Mohamed Hussein, Nadia M. ElRouby

**Affiliations:** 1 Basic Sciences Department, College of Medicine, RAK Medical and Health Sciences University, Ras Al Khaimah 11172, United Arab Emirates; 2 Biomedical Science Department, Dubai Medical College for Girls, Dubai Medical University, Dubai 20170, United Arab Emirates; dr.sarah@dmu.ae (S.S.); or nadia61mohd@gmail.com (N.M.E.); 3 Neurology Department, Zulekha Hospital, Dubai 48577, United Arab Emirates; dr.link147@yahoo.com (M.E.); loomy16@yahoo.com (L.I.); myounan@zulekhahospital.com (M.A.L.Y.); shyambabuc@gmail.com (S.B.C.)

**Keywords:** migraine, ACE I/D polymorphism, genetic association, neurovascular mechanisms, renin–angiotensin system, genotype–phenotype relationship, precision medicine, UAE population

## Abstract

**Background/Objectives:** Migraine is a complex neurovascular disorder with a multifactorial genetic basis and remains a leading cause of neurological disability worldwide. The angiotensin-converting enzyme (ACE) insertion/deletion (I/D) polymorphism has been implicated in vascular regulation and neurovascular reactivity; however, its role in migraine susceptibility and clinical expression remains unclear. This study aimed to evaluate the association between ACE I/D polymorphism and migraine susceptibility and clinical phenotypes in a diverse clinical cohort. **Methods:** A case–control study was conducted including 183 participants, comprising clinically diagnosed migraine patients (*n* = 82) and age- and sex-matched controls (*n* = 101). Genomic DNA was extracted from peripheral blood samples, and ACE I/D genotyping was performed using polymerase chain reaction. Genotype distribution was compared using chi-square tests, and odds ratios (ORs) with 95% confidence intervals (CIs) were calculated. **Results:** Overall genotype frequencies were Deletion/Deletion (DD) 43.7%, ID 38.8%, and Insertion/Insertion (II) 17.5%. Genotype distributions were comparable between migraine patients and controls (χ^2^ ≈ 0.92, *p* = 0.632). No statistically significant associations were identified between ACE I/D polymorphism and migraine susceptibility, genotype models, or migraine subtypes. However, the II genotype was associated with higher Migraine Disability Assessment (MIDAS) scores among migraine patients. **Conclusions:** The ACE I/D polymorphism was not associated with migraine susceptibility or clinical phenotypes in this cohort. These findings suggest that ACE I/D polymorphism does not appear to be a major determinant of migraine susceptibility in this cohort. The results highlight the need to investigate broader genetic and molecular mechanisms and support the application of integrative genomic approaches to better understand migraine heterogeneity and inform precision medicine strategies.

## 1. Introduction

Migraine is a highly prevalent and disabling neurological disorder, affecting approximately 14–15% of the global population and representing one of the leading causes of years lived with disability worldwide [1,2]. Clinically, it is characterized by recurrent attacks of moderate to severe headache often accompanied by sensory disturbances, autonomic symptoms, and significant functional impairment. The underlying pathophysiology of migraine is complex and multifactorial, involving complex interactions among neurovascular, neuroinflammatory, and neuromodulatory mechanisms that contribute to both the initiation and propagation of migraine attacks [3].

From a clinical perspective, migraine-related disability is commonly assessed using the Migraine Disability Assessment (MIDAS) questionnaire, a validated instrument that quantifies functional impairment in relation to headache frequency and severity [4]. The incorporation of disability measures such as MIDAS provides essential clinical context, enabling a more comprehensive evaluation of disease burden beyond symptom description alone [5].

Migraine epidemiology is further shaped by pronounced sex- and age-related differences. Women experience migraine nearly three times more frequently than men, a disparity largely attributed to hormonal influences, particularly fluctuations in estrogen, which modulate cortical excitability, trigeminovascular activation, and central pain processing pathways [6,7]. In addition, migraine prevalence follows a characteristic age-related trajectory, typically increasing during adolescence, peaking between 30 and 40 years of age, and gradually declining thereafter [8]. These demographic patterns underscore the importance of biological and hormonal factors in migraine expression and highlight the need to account for age- and sex-specific influences when investigating potential genetic determinants.

While environmental triggers and hormonal factors are well-recognized contributors to migraine attacks, a substantial body of evidence supports a strong genetic predisposition that influences both disease susceptibility and clinical heterogeneity [9,10]. In recent years, increasing attention has been directed toward candidate genes involved in vascular regulation and neurovascular coupling. Among these, the angiotensin-converting enzyme (ACE) gene has emerged as a biologically plausible candidate due to its central role in the renin–angiotensin aldosterone system (RAAS), which regulates vascular tone, endothelial function, and cerebral blood flow processes implicated in migraine pathophysiology [11,12].

The ACE gene is located on chromosome 17q23, spans approximately 21 kilobases, and comprises 26 exons and 25 introns. The most extensively studied variant is a 287–base pair insertion/deletion (I/D) polymorphism in intron 16, resulting in three genotypes: II, ID, and DD. This polymorphism is functionally relevant, as the deletion (D) allele is associated with higher circulating and tissue ACE activity, potentially influencing cerebrovascular reactivity, endothelial responses, and nociceptive signaling pathways. Consequently, the ACE I/D polymorphism has been investigated in relation to migraine susceptibility and phenotypic variability; however, findings across populations remain inconsistent [13,14].

Recent advances in migraine research have increasingly emphasized the role of complex genetic architectures and polygenic risk models in disease susceptibility. Genome-wide association studies (GWAS) have identified more than 100 genetic loci associated with migraine, highlighting its polygenic nature and the contribution of diverse biological pathways, including vascular regulation, ion channel function, and neurotransmitter signaling [9,15]. Furthermore, emerging evidence supports the integration of genetic findings with clinical phenotyping to improve disease stratification and inform precision medicine approaches. Despite these advances, the clinical utility of individual candidate gene polymorphisms remains limited, underscoring the need for continued evaluation across diverse populations.

Despite the biological plausibility of the ACE I/D polymorphism as a candidate genetic factor in migraine and a growing body of international evidence, findings across different populations remain inconsistent [16]. While some studies have reported associations between ACE I/D variants and migraine susceptibility or clinical characteristics, others have found no significant relationship. In addition, data from Middle Eastern populations, including the United Arab Emirates, remain limited despite the potential influence of population-specific genetic and demographic factors. Given these inconsistencies and the scarcity of regional evidence, further investigation is warranted. Accordingly, the present study aimed to evaluate the association between ACE I/D polymorphism and migraine susceptibility and its relationship with clinical migraine phenotypes in a well-characterized UAE cohort. By addressing this gap, this study contributes population-specific evidence while helping to contextualize candidate gene findings within the evolving landscape of migraine genetics and precision medicine.

## 2. Materials and Methods

### 2.1. Study Design and Population

This case–control genetic association study was conducted at a neurology clinic in Dubai, United Arab Emirates. Migraine cases were recruited consecutively from patients attending the neurology clinic during the study period. Eligible participants were adults aged 18 years or older with a confirmed diagnosis of migraine established by consultant neurologists according to the International Classification of Headache Disorders, 3rd edition (ICHD-3) criteria [17]. Both episodic and chronic migraine patients were eligible for inclusion. Inclusion criteria for the migraine group were: (1) age ≥ 18 years and (2) a confirmed clinical diagnosis of migraine according to ICHD-3 criteria. Exclusion criteria included inability to provide informed consent, presence of significant neurological disorders other than migraine, or unavailable genotyping data. Control participants were recruited from individuals attending the same healthcare setting or accompanying patients during the study period. Controls were frequency matched to migraine cases by age and sex whenever possible. Eligibility required the absence of a previous diagnosis of migraine and no history suggestive of migraine according to screening questions administered during participant assessment. Individuals reporting recurrent headaches, physician-diagnosed migraine, or other primary headache disorders were not eligible for inclusion in the control group. All potential controls were asked about a history of migraine, recurrent headaches, and previous neurological diagnoses before enrollment Standardized data collection and matching procedures were applied to minimize potential sources of selection and information bias. The study was reported in accordance with the STROBE guidelines for observational studies.

The sample consisted of all eligible participants recruited during the study period at the neurology clinic. All individuals meeting the inclusion criteria were consecutively recruited. A formal sample size calculation was not undertaken because all eligible participants were recruited consecutively, and the sample was considered adequate for exploratory genetic association analysis. Participants with incomplete genotyping data were excluded from the final analysis.

The primary outcome was migraine status (case versus control), with secondary analyses examining migraine subtypes (episodic and chronic). The primary exposure variable was ACE I/D genotype (II, ID, DD). Age and sex were considered potential confounders. Migraine diagnoses were established by experienced neurologists according to the International Classification of Headache Disorders, 3rd edition (ICHD-3) criteria.

### 2.2. Ethical Considerations

The study was approved by the Research Ethics Committee of Dubai Medical College for Girls, United Arab Emirates (Approval No. REC/DMCG/AY21-22/F-000; approved on 31 May 2022). Written informed consent was obtained from all participants prior to enrollment and blood sample collection. All assessment procedures, including clinical evaluation, laboratory analysis, and data collection, were applied uniformly across cases and controls to ensure comparability between groups.

### 2.3. Sample Collection and DNA Extraction

Venous blood was collected in EDTA tubes, and genomic DNA was isolated using standard extraction procedures. DNA samples were stored at −20 °C until genotyping.

### 2.4. Genotyping

The ACE I/D polymorphism was identified by polymerase chain reaction (PCR) amplification targeting intron 16. PCR products were resolved by agarose gel electrophoresis, yielding bands corresponding to the insertion (490 bp), deletion (190 bp), or both (heterozygous ID).

To genotype insertion/deletion (I/D) polymorphisms in the angiotensin-converting enzyme (ACE) gene, PCR amplification targeting the 16th intron, where a 287 bp Alu repeat distinguishes the I allele from the D allele, was performed using a GenAmp^®^ 9700 thermal cycler (Applied Biosystems, Foster City, CA, USA). Each 25 μL reaction contained 10 pmol of forward (5′-CTGGAGACCACTCCCATCCTTTCT-3′) and reverse (5′-GATGTGGCCATCACATTCGTCAGAT-3′) primers, 1.5 mM MgCl_2_, 0.2 mM of each dNTP (Vivantis Technologies Sdn. Bhd., Selangor, Malaysia), 1 unit of hot-start Taq DNA polymerase (Vivantis Technologies Sdn. Bhd., Selangor, Malaysia), 2 μL of genomic DNA (50–100 ng/μL) extracted using the NucleoSpin^®^ Blood Kit (Takara Bio Inc., Kusatsu, Japan), and nuclease-free water to complete the volume. The mixture was gently vortexed and centrifuged before amplification under the following thermal cycling conditions: initial denaturation at 95 °C for 2 min; 30 cycles of denaturation at 94 °C for 30 s, annealing at 58 °C for 30 s, and extension at 72 °C for 45 s; followed by a final extension at 72 °C for 9 min. Amplified products were held at 4 °C until analysis. PCR yielded a 490 bp fragment for the insertion (I) allele and a 190 bp fragment for the deletion (D) allele; genotypes were identified based on agarose gel electrophoresis banding patterns.

**Figure 1:**
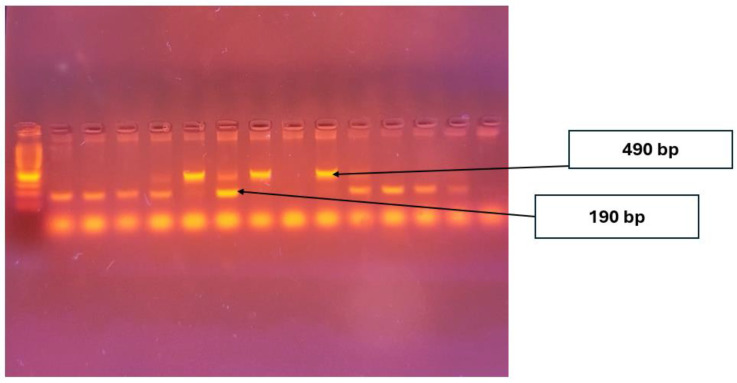
Agarose gel electrophoresis image of PCR-amplified ACE I/D polymorphism showing insertion (490 bp) and deletion (190 bp) alleles. The Colors shown in the gel image are due to the DNA staining and the UV visualization process and do not represent different experimental groups or variable.

All PCR reactions were performed using standardized laboratory protocols with appropriate reagent preparation and handling procedures. Amplification products were evaluated by agarose gel electrophoresis, and genotype assignments were based on clearly identifiable banding patterns corresponding to the II, ID, and DD genotypes. Samples with unclear or ambiguous electrophoretic patterns were re-evaluated and repeated when necessary to ensure accurate genotype classification. ACE I/D genotyping was performed according to the method described by Rigat et al. with minor modifications [18]. Representative agarose gel electrophoresis results of the ACE I/D PCR products are shown in Figure 1.

### 2.5. Clinical Assessment

Migraine-related disability was assessed using the Migraine Disability Assessment (MIDAS) questionnaire. MIDAS scores were available for 80 migraine patients, while two cases had missing data and were excluded from MIDAS-related analyses.

Migraine patients were additionally classified into episodic and chronic migraine subtypes according to established clinical criteria.

### 2.6. Statistical Analysis

Statistical analyses were performed using SPSS version 29 (IBM Corp., Armonk, NY, USA). Continuous variables were expressed as mean ± standard deviation (SD) or median (interquartile range [IQR]), as appropriate according to data distribution. Categorical variables were summarized as frequencies and percentages.

Comparisons between migraine patients and controls were performed using the independent samples *t*-test for continuous variables and chi-square test or Fisher’s exact test for categorical variables, as appropriate. Odds ratios (ORs) with 95% confidence intervals (CIs) were calculated to estimate the association between ACE I/D polymorphism and migraine susceptibility.

Additionally, multivariable logistic regression analysis adjusting for age and sex was performed to assess the robustness of the association between ACE I/D polymorphism and migraine susceptibility. The results are presented in Supplementary Table S1.

Hardy–Weinberg equilibrium was assessed separately in migraine patients and controls using chi-square analysis. MIDAS scores across genotype groups were compared using the Kruskal–Wallis test. A *p*-value < 0.05 was considered statistically significant. One participant had incomplete age-specific subgroup data and was excluded from selected subgroup analyses where applicable.

## 3. Results

### 3.1. Sociodemographic Characteristics

A total of 183 participants were included in the final analysis, comprising 82 clinically diagnosed migraine patients and 101 control subjects. The mean age was 37.5 ± 7.6 years in migraine patients and 39.6 ± 11.2 years in controls, with no statistically significant difference between groups (*t* = −1.47, *p* = 0.144). Females represented the majority of migraine patients (75.6%) and were significantly more frequent in the migraine group compared with controls (χ^2^ = 5.85, *p* = 0.016). No statistically significant differences were observed between migraine patients and controls with respect to ethnic background (χ^2^ = 1.02, *p* = 0.311). The largest proportion of migraine patients belonged to the 31–40-year age category (59.3%). Among migraine patients, episodic migraine represented 90.2% of cases, whereas chronic migraine accounted for 9.8%. Sociodemographic and clinical characteristics are summarized in Table 1.

### 3.2. Age and Sex Distribution of Migraine Patients

The genotype and allele distributions of ACE I/D polymorphism among migraine patients and controls are presented in Table 2. Overall genotype frequencies in the study cohort were DD 43.7%, ID 38.8%, and II 17.5%.

No statistically significant differences were observed in genotype distribution between migraine patients and controls (χ^2^ = 0.92, *p* = 0.632). Using the II genotype as the reference category, the ID genotype demonstrated an OR of 1.07 (95% CI: 0.46–2.50, *p* = 1.000), while the DD genotype demonstrated an OR of 1.39 (95% CI: 0.61–3.19, *p* = 0.531).

Similarly, neither the dominant nor the recessive genetic model demonstrated an association with migraine susceptibility. Under the dominant model (DD + ID vs. II), the OR was 1.23 (95% CI: 0.57–2.67, *p* = 0.697). Under the recessive model (DD vs. ID + II), the OR was 1.33 (95% CI: 0.74–2.39, *p* = 0.371).

Allele frequency analysis also demonstrated no statistically significant association with migraine susceptibility. The D allele frequency was 65.9% among migraine patients and 60.9% among controls (χ^2^ = 0.76, *p* = 0.385), corresponding to an OR of 1.24 (95% CI: 0.81–1.90). Overall, no genotype, allele, or inheritance model comparisons were statistically significant. To account for potential confounding, an additional multivariable logistic regression analysis adjusting for age and sex was performed. The adjusted findings were consistent with the primary analyses and did not materially alter the observed association between ACE I/D polymorphism and migraine susceptibility (Supplementary Table S1).

### 3.3. Genotype Distribution and Association with Migraine

The distribution of ACE I/D genotypes and allele frequencies across migraine subtypes is presented in Table 3. Episodic migraine accounted for 74 cases, whereas chronic migraine included 8 cases.

Among episodic migraine patients, the DD genotype represented the predominant genotype (50.0%), followed by ID (37.8%) and II (12.2%). In contrast, chronic migraine patients demonstrated that the II genotype occurred more frequently among chronic migraine cases, a (50.0%) compared with episodic migraine patients (12.2%) and controls (18.8%).

Comparison between episodic migraine patients and controls demonstrated no statistically significant difference in genotype distribution (χ^2^ = 2.11, *p* = 0.348). Although differences in allele frequencies were observed descriptively, the chronic migraine subgroup size was too small to permit reliable statistical comparison. Therefore, findings related to chronic migraine genotype distribution should be interpreted cautiously.

### 3.4. Hardy–Weinberg Equilibrium Assessment

Hardy–Weinberg equilibrium analysis was performed separately for migraine patients and controls. Neither group demonstrated significant deviation from equilibrium, supporting the validity and reliability of the genotyping data.

Among controls, the genotype distribution was consistent with Hardy–Weinberg equilibrium expectations (χ^2^ = 2.20, *p* = 0.138). Similarly, migraine patients showed no significant deviation from equilibrium (χ^2^ = 2.85, *p* = 0.091). The observed and expected genotype frequencies are presented in Table 4.

### 3.5. ACE I/D Genotype Distribution According to Sex

Sex-stratified genotype analysis demonstrated no statistically significant differences in ACE I/D genotype distribution between migraine patients and controls within either sex category.

Among females, genotype distributions did not differ significantly between migraine patients and controls (χ^2^ = 0.496). Similarly, no statistically significant differences were observed among males (χ^2^ = 0.903). Detailed genotype frequencies according to sex are presented in Table 5.

### 3.6. ACE I/D Genotype Distribution According to Ethnic Background

Genotype distributions stratified according to ethnic background are presented in Table 6. No statistically significant differences in ACE I/D genotype distribution were identified between migraine patients and controls within either Arab or non-Arab subgroups.

Among Arab participants, genotype distributions were comparable between migraine patients and controls (χ^2^ = 0.859). Similarly, no statistically significant differences were identified among non-Arab participants (χ^2^ = 0.202) (Table 6).

### 3.7. MIDAS Scores According to ACE I/D Genotype

MIDAS disability scores according to ACE I/D genotype are presented in Table 7. Two migraine patients had missing MIDAS data and were excluded from MIDAS-related analyses, resulting in a final sample of 80 migraine patients.

A statistically significant difference in MIDAS scores was observed across genotype groups (Kruskal–Wallis H = 6.28, df = 2, *p* = 0.043). Patients carrying the II genotype demonstrated the highest migraine-related disability scores (mean ± SD: 12.3 ± 6.0; median [IQR]: 14.0 [8.0–18.0]) compared with the DD genotype (7.9 ± 4.0; median 8.0 [4.0–10.0]) and ID genotype (8.6 ± 4.9; median 9.5 [4.0–12.0]).

These findings suggest that although ACE I/D polymorphism was not associated with migraine susceptibility, genotype-related differences may influence migraine-related disability severity (Table 5).

## 4. Discussion

This study evaluated the association between ACE I/D genetic variants and migraine susceptibility and clinical phenotypes. No statistically significant differences were observed in genotype or allele distributions between migraine patients and controls, and no significant associations were identified across migraine subtypes. However, migraine-related disability scores differed significantly across ACE I/D genotypes, with the II genotype associated with higher MIDAS disability scores. MIDAS scores were broadly consistent with those reported in outpatient migraine populations, supporting the clinical representativeness of the sample and strengthening the interpretation of genotype–phenotype relationships.

These findings are consistent with several large-scale studies conducted across Europe, North America, and Asia, which have similarly reported no robust association between the ACE I/D polymorphism and migraine susceptibility [5,19,20]. More recent evidence further supports the limited influence of genetic susceptibility markers on migraine clinical expression. For instance, a contemporary community-based cohort study demonstrated that genetic susceptibility scores do not strongly influence migraine characteristics or clinical outcomes, reinforcing the notion that individual genetic variants may have limited phenotypic impact in real-world settings [21]. Together, the findings of this study support the view that the ACE I/D polymorphism has limited influence on migraine susceptibility in the present cohort. While no significant association was observed in this sample, future studies involving larger populations may further clarify the extent to which ACE-related genetic variation contributes to migraine risk and clinical heterogeneity.

Although no significant association with migraine susceptibility was observed, these findings contribute to the accumulating evidence suggesting that ACE I/D polymorphism is unlikely to be a major genetic determinant of migraine risk. Interestingly, patients carrying the II genotype demonstrated higher MIDAS disability scores, suggesting a possible genotype–phenotype relationship. While this finding should be interpreted cautiously given the relatively modest sample size, it raises the possibility that ACE-related pathways are more closely associated with migraine severity than susceptibility. Further studies involving larger cohorts are warranted to validate this observation and explore its potential clinical implications. Importantly, the consistency of the findings following adjustment for age and sex further supports the robustness of the primary analysis.

Beyond migraine, ACE I/D polymorphism has been extensively studied in other clinical and physiological contexts, including cardiovascular disease, pulmonary outcomes, and physical performance. Recent studies have shown associations between ACE polymorphisms and susceptibility to conditions such as hypertension and COVID-19-related complications, reflecting its role in systemic vascular and inflammatory regulation [22,23]. Similarly, evidence from sports medicine indicates that ACE I/D variation may influence physical performance and injury risk, although findings remain inconsistent and context-dependent [24,25]. These observations highlight the biological relevance of ACE within the renin–angiotensin system; however, they also underscore that its effects are highly variable across conditions and populations and may not directly translate to migraine susceptibility.

From a mechanistic perspective, ACE is a key component of the renin–angiotensin system (RAAS), which regulates vascular tone, endothelial function, and neurovascular coupling-processes implicated in migraine pathophysiology [26,27]. It has been hypothesized that increased ACE activity associated with the D allele may influence cerebrovascular reactivity and nociceptive signaling. However, the absence of association observed in this study suggests that ACE I/D polymorphism alone is insufficient to account for the complex neurovascular mechanisms underlying migraine. These findings further support the current understanding of migraine as a multifactorial and polygenic disorder, in which the contribution of individual candidate genes is likely modest and context-dependent [28,29].

When considered alongside evidence from meta-analyses and large cohort studies, the present findings further indicate that ACE I/D polymorphism has limited clinical and biological relevance in migraine [7,20]. These observations highlight the limitations of single candidate gene approaches and support a shift toward integrative genomic strategies capable of capturing the complex and heterogeneous nature of migraine susceptibility.

From a clinical and translational perspective, these findings have implications for precision medicine. Although ACE I/D polymorphism was not associated with migraine susceptibility, the observed genotype-related differences in MIDAS disability scores suggest a potential influence on disease severity and functional burden. While the current findings do not support the use of ACE I/D polymorphism as a reliable biomarker for migraine susceptibility, further research may help clarify whether this variant contributes to clinical heterogeneity or patient stratification in migraine. Future studies should therefore focus on multi-locus genetic models and integrated approaches combining genomic, environmental, and clinical data to improve personalized management strategies in migraine.

This study has several strengths, including rigorous clinical characterization of migraine cases, laboratory-confirmed genotyping, and a matched case–control design that minimizes potential confounding. The inclusion of a multiethnic cohort enhances the relevance of the findings within an underrepresented population. However, several limitations should be acknowledged. The relatively modest sample size, particularly for the migraine group (*n* = 82) and the chronic migraine subgroup (*n* = 8), may have limited the statistical power to detect modest genetic effects and subtle genotype–phenotype associations, increasing the possibility of type II error. Consequently, findings related to migraine subtypes should be interpreted with caution. Additionally, the absence of detailed aura classification limited more granular phenotypic analyses, and the evaluation of a single candidate polymorphism provides only a limited perspective on the complex polygenic architecture of migraine. Although controls reported no personal history of migraine, formal headache-specific screening instruments were not administered; therefore, the possibility of unrecognized headache disorders cannot be completely excluded. Finally, the single-center design may limit the generalizability of the findings to broader populations. Future multicenter studies with larger sample sizes and more comprehensive genetic profiling are warranted to validate and extend these findings.

Collectively, these findings underscore the importance of validating genetic associations across diverse populations and highlight the need to move beyond single-gene approaches in the study of complex disorders such as migraine. Future research should prioritize larger, multi-center investigations and adopt comprehensive genomic and multi-omics approaches, including polygenic risk modeling, to better elucidate the genetic determinants of migraine susceptibility and clinical heterogeneity. Such approaches are essential to advance precision medicine in migraine, enabling more accurate risk stratification and individualized therapeutic interventions.

## 5. Conclusions

In this clinical–genetic study, ACE I/D polymorphism was not identified as a significant determinant of migraine susceptibility in this UAE cohort. Although no significant associations were identified across migraine subtypes, the II genotype was associated with higher migraine-related disability scores, suggesting a possible relationship with disease severity rather than susceptibility. These findings are consistent with the multifactorial and polygenic nature of migraine and support the need for broader genomic and multi-omics approaches to better characterize migraine heterogeneity and advance precision medicine strategies, particularly in underrepresented populations.

## Figures and Tables

**Table 1:** Sociodemographic and clinical characteristics of migraine patients and controls (*n* = 183).

Variable	Migraine (*n* = 82)	Control (*n* = 101)	χ^2^/*t*	*p*
Age, M (SD)	37.5 (7.6)	39.6 (11.2)	*t* = −1.47	0.144
Sex, *n* (%)			χ^2^ = 5.85	0.016 *
Female	62 (75.6)	58 (57.4)		
Male	20 (24.4)	43 (42.6)		
Ethnic background, *n* (%)			χ^2^ = 1.02	0.311
Arab	45 (54.9)	64 (63.4)		
Non-Arab	37 (45.1)	37 (36.6)		
Age category, *n* (%)				
18–30 years	11 (13.6)	24 (23.8)		
31–40 years	48 (59.3)	34 (33.7)		
41–50 years	17 (21.0)	27 (26.7)		
>51 years	5 (6.2)	16 (15.8)		
Migraine classification, *n* (%)				
Episodic	74 (90.2)	—		
Chronic	8 (9.8)	—		

Note. M = mean; SD = standard deviation. Age comparison by independent-samples *t*-test; sex and ethnicity by χ^2^ test. One participant had missing age data (*n* = 182 for the age category). MIDAS scores were available for 80 migraine patients; two participants had missing MIDAS data and were excluded from MIDAS-related analyses. Percentages for migraine age categories based on *n* = 81. * *p* < 0.05.

**Table 2:** Association of ACE I/D genotype and allele frequency with migraine status (*n* = 183).

	Migraine (*n* = 82)	Control (*n* = 101)	OR	95% CI	*p*	χ^2^	*p* (Overall)
**Genotype, *n* (%)**						0.92	0.632
II (reference)	13 (15.9)	19 (18.8)	1.00	—	—		
ID	30 (36.6)	41 (40.6)	1.07	[0.46, 2.50]	1.000		
DD	39 (47.6)	41 (40.6)	1.39	[0.61, 3.19]	0.531		
**Genetic model**							
Dominant (DD + ID vs. II)	69 (84.1)	82 (81.2)	1.23	[0.57, 2.67]	0.697		
Recessive (DD vs. ID + II)	39 (47.6)	41 (40.6)	1.33	[0.74, 2.39]	0.371		
**Allele, *n* (%)**						0.76	0.385
I (reference)	56 (34.1)	79 (39.1)	1.00	—	—		
D	108 (65.9)	123 (60.9)	1.24	[0.81, 1.90]	0.385		

Note. OR = odds ratio; CI = confidence interval. Genotype ORs calculated using II as reference via 2 × 2 Fisher’s exact test. Genetic model ORs from Fisher’s exact test. Allele comparison by χ^2^ test. No comparisons reached statistical significance.

**Table 3:** Distribution of ACE I/D genotypes and allele frequencies across migraine subtypes.

Genotype, *n* (%)	Control (*n* = 101)	Episodic (*n* = 74)	Chronic (*n* = 8)	χ^2^	*p*
DD	41 (40.6)	37 (50.0)	2 (25.0)		
ID	41 (40.6)	28 (37.8)	2 (25.0)		
II	19 (18.8)	9 (12.2)	4 (50.0)		
Episodic vs. Control				2.11	0.348
**Allele frequency, *n* (%)**					
D	123 (60.9)	102 (68.9)	6 (37.5)		
I	79 (39.1)	46 (31.1)	10 (62.5)		

Note. Episodic vs. control comparison by 3 × 2 χ^2^ test. Chronic subgroup (*n* = 8) was too small for reliable statistical comparison. A higher proportion of II genotype was observed in chronic migraine (50.0%) relative to episodic (12.2%) and controls (18.8%); however, this should be interpreted cautiously given the small sample size.

**Table 4:** Hardy–Weinberg equilibrium assessment for ACE I/D genotype distribution.

Group	Observed			Expected		
	DD	ID	II	DD	ID	II
Controls (χ^2^ = 2.20, *p* = 0.138)	41	41	19	37.4	48.1	15.4
Migraine (χ^2^ = 2.85, *p* = 0.091)	39	30	13	35.6	36.9	9.6

Note. Expected frequencies calculated under Hardy–Weinberg equilibrium assumptions. χ^2^ test with *df* = 1. Neither group deviated significantly from equilibrium, supporting the validity of genotyping data.

**Table 5:** ACE I/D genotype distribution by sex in migraine patients and controls.

Genotype	Females			Males		
	Migraine	Control	*p*	Migraine	Control	*p*
DD, *n* (%)	29 (46.8)	21 (36.2)		10 (50.0)	20 (46.5)	
ID, *n* (%)	22 (35.5)	24 (41.4)		8 (40.0)	17 (39.5)	
II, *n* (%)	11 (17.7)	13 (22.4)		2 (10.0)	6 (14.0)	
χ^2^ (overall)			0.496			0.903

Note. *p* values from 3 × 2 χ^2^ test comparing genotype distribution between migraine and control groups within each sex. No statistically significant differences were observed.

**Table 6:** ACE I/D genotype distribution by ethnic background in migraine patients and controls.

Genotype	Arab			Non-Arab		
	Migraine	Control	*p*	Migraine	Control	*p*
DD, *n* (%)	24 (53.3)	33 (51.6)		15 (40.5)	8 (21.6)	
ID, *n* (%)	15 (33.3)	20 (31.3)		15 (40.5)	21 (56.8)	
II, *n* (%)	6 (13.3)	11 (17.2)		7 (18.9)	8 (21.6)	
χ^2^ (overall)			0.859			0.202

Note. *p* values from 3 × 2 χ^2^ test comparing genotype distribution between migraine and control groups within each ethnic stratum. Arab classification based on nationality. No statistically significant differences were observed.

**Table 7:** MIDAS Scores by ACE I/D Genotype Among Migraine Patients (*n* = 80).

Genotype	*n*	M (SD)	Mdn (IQR)	*p*
DD	37	7.9 (4.0)	8.0 (4.0–10.0)	
ID	30	8.6 (4.9)	9.5 (4.0–12.0)	
II	13	12.3 (6.0)	14.0 (8.0–18.0)	0.043 *

Note. M = mean; SD = standard deviation; Mdn = median; IQR = interquartile range. Two participants had missing MIDAS data. *p* value from Kruskal–Wallis *H* test (*H* = 6.28, *df* = 2). The II genotype was associated with higher MIDAS scores, suggesting greater migraine-related disability. * *p* < 0.05.

## Data Availability

The original contributions presented in the study are included in the article/Supplementary Material; further inquiries can be directed to the corresponding authors.

## Supplementary Materials

The following supporting information can be downloaded at: https://www.mdpi.com/article/10.3390/jcm15145748/s1, Table S1: Hierarchical binary logistic regression predicting self-medication with analgesics (*n* = 400). Table S2: Hierarchical multiple linear regression predicting analgesic safety knowledge score (*n* = 332). Table S3: Binary logistic regression predicting adequate analgesic safety knowledge (≥4/5 correct; *n* = 332). Table S4: Age- and sex-adjusted odds ratios for correct response on individual knowledge items (*n* = 332).

## Abbreviations

The following abbreviations are used in this manuscript:

ACE	angiotensin-converting enzyme
ICHD-3	International Classification of Headache Disorders, 3rd edition criteria
I/D	insertion/deletion
I/I	insertion/insertion
D/D	deletion/deletion
MIDAS	Migraine Disability Assessment (MIDAS)
RAAS	renin–angiotensin system
