# Supplementary material for: Angiotensin-Converting Enzyme (ACE) Insertion/Deletion (I/D) Polymorphism and Migraine Susceptibility and Phenotypes: A Clinical-Genetic Study in a United Arab Emirates (UAE) Cohort
Source: J Clin Med. 2026 Jul 22;15(14):5748. doi: 10.3390/jcm15145748 (PMC13412727; doi:10.3390/jcm15145748)
Supplement: Supplementary file 1 [file jcm-15-05748-s001.zip › jcm-4360879-supplementary.pdf]

Table S1 presents logistic regression models predicting self-medication; Table S2 presents linear regression models predicting the continuous knowledge score; Table S3 presents a logistic regression predicting adequate knowledge (≥4/5 correct); and Table S4 reports age- and sex-adjusted odds ratios for each individual knowledge item.

**Table S1**

*Hierarchical binary logistic regression predicting self-medication with analgesics (n = 400)*

| | | | | | | |
|---|---|---|---|---|---|---|
| ***Model 1: Age + sex*** | | | | | | |
| Constant | 0.357 | 0.537 | 0.443 | .506 | 1.429 | [0.499, 4.092] |
| Sex (female) | 0.716 | 0.314 | 5.212 | .022* | 2.047 | [1.107, 3.786] |
| Age | 0.023 | 0.012 | 3.652 | .056 | 1.023 | [0.999, 1.048] |
| ***Model 2: Age + sex + education*** | | | | | | |
| Constant | −0.758 | 1.000 | 0.575 | .449 | 0.469 | [0.066, 3.327] |
| Sex (female) | 0.733 | 0.315 | 5.415 | .020* | 2.082 | [1.123, 3.862] |
| Age | 0.018 | 0.013 | 2.187 | .139 | 1.019 | [0.994, 1.044] |
| Education level | 0.446 | 0.343 | 1.696 | .193 | 1.563 | [0.798, 3.059] |

*Note.* Sex coded 0 = male, 1 = female. Age entered as continuous (estimated category midpoints). Education coded as ordinal (1 = primary, 2 = secondary, 3 = university+). Model 1: $\chi^2(2) = 6.16$, $p = .046$; Nagelkerke $R^2 = .026$; Hosmer–Lemeshow $\chi^2(5) = 5.04$, $p = .411$. Model 2: Nagelkerke $R^2 = .032$; likelihood ratio test for addition of education: $\chi^2(1) = 1.63$, $p = .202$. Classification accuracy: 83.0% in both models. *$p < .05$.

After adjusting for age, female sex was independently associated with approximately twice the odds of self-medication (Model 1: OR = 2.05, 95% CI [1.11, 3.79], $p = .022$). Age showed a marginal positive trend (OR = 1.02 per year, $p = .056$), suggesting a tendency toward higher self-medication prevalence with increasing age that did not reach conventional significance. The parsimonious age + sex model was statistically significant ($\chi^2(2) = 6.16$, $p = .046$), with adequate fit (Hosmer–Lemeshow $p = .411$).

Adding education to the model (Model 2) did not significantly improve fit (likelihood ratio $\chi^2(1) = 1.63$, $p = .202$; ΔNagelkerke $R^2 = .007$). This confirms that education level contributed no meaningful independent explanatory value beyond age and sex. Of note, the age coefficient was attenuated in Model 2 (OR 1.02 → 1.02, $p = .056 \rightarrow .139$), suggesting partial collinearity with education. Both models explained only modest variance (Nagelkerke $R^2 = .026$–$.032$), consistent with the bivariate findings and with self-medication being a normative behavior largely independent of standard demographic predictors in this population.

**Table S2**

*Hierarchical Multiple Linear Regression Predicting Analgesic Safety Knowledge Score (n = 332)*

| Predictor | *B* | *SE* | *t* | *p* | β | *sr²* | 95% CI |
|---|---|---|---|---|---|---|---|
| ***Model 1: Age + sex*** | | | | | | | |
| Constant | 3.835 | 0.341 | 11.250 | <.001*** | — | | [3.164, 4.505] |
| Sex (female) | 0.409 | 0.200 | 2.047 | .041* | .192 | .012 | [0.016, 0.802] |
| Age | −0.031 | 0.007 | −4.321 | <.001*** | –.406 | .052 | [−0.045, −0.017] |
| ***Model 2: Age + sex + education*** | | | | | | | |
| Constant | 2.960 | 0.729 | 4.062 | <.001*** | — | | [1.526, 4.393] |
| Sex (female) | 0.427 | 0.200 | 2.135 | .034* | .201 | .012 | [0.033, 0.820] |
| Age | −0.033 | 0.007 | −4.528 | <.001*** | –.439 | .055 | [−0.047, −0.019] |
| Education | 0.332 | 0.245 | 1.359 | .175 | .120 | .005 | [−0.149, 0.813] |

*Note.* β = standardized coefficient. $sr^2$ = squared semi-partial correlation. Model 1: $R^2 = .105$, adjusted $R^2 = .099$, $F(2, 329) = 19.26$, $p < .001$. Model 2: $R^2 = .110$, adjusted $R^2 = .102$, $F(3, 328) = 13.49$, $p < .001$; $\Delta R^2 = .005$, $p = .175$. *$p < .05$. ***$p < .001$.

In the primary model adjusting for age and sex, the overall regression was significant, $F(2, 329) = 19.26$, $p < .001$, accounting for 10.5% of variance in knowledge scores. Age was the dominant predictor (β = –.406, $p < .001$), uniquely explaining 5.2% of variance: each 10-year increase in estimated age was associated with a 0.31-point decline in knowledge score. Female sex was independently associated with higher knowledge (β = .192, $p = .041$), with an adjusted mean difference of 0.41 points.

The addition of education level (Model 2) yielded a non-significant increment in explained variance ($\Delta R^2 = .005$, $p = .175$), and the age and sex coefficients remained substantively unchanged. A gender × age interaction term was tested but was non-significant ($\Delta R^2 < .001$, $p = .916$, not shown), confirming that the age-related knowledge decline operated similarly in both sexes. These results demonstrate that older age is the most robust independent predictor of lower analgesic safety knowledge, with female sex providing a smaller but significant protective effect that persists after adjustment.

**Table S3**

*Binary Logistic Regression Predicting Adequate Analgesic Safety Knowledge (≥4/5 Correct; n = 332)*

| Predictor | *B* | *SE* | Wald | *p* | OR | 95% CI |
|---|---|---|---|---|---|---|
| Constant | 1.051 | 0.472 | 4.956 | .026* | 2.859 | [1.134, 7.209] |
| Sex (female) | 0.422 | 0.278 | 2.293 | .130 | 1.524 | [0.883, 2.631] |
| Age | −0.046 | 0.010 | 19.903 | <.001*** | 0.955 | [0.936, 0.975] |

*Note.* Adequate knowledge defined as ≥4 of 5 items correct (44.3% of self-medicators). Model $\chi^2(2) = 36.41$, $p < .001$. Nagelkerke $R^2 = .139$. Classification accuracy: 66.3% (sensitivity: 61.9%; specificity: 69.7%). *$p < .05$. ***$p < .001$.

To complement the continuous analysis, a binary logistic regression was conducted predicting adequate knowledge (≥4/5 correct) adjusted for age and sex. The model was highly significant ($\chi^2(2) = 36.41$, $p < .001$; Nagelkerke $R^2 = .139$). Age was the sole significant predictor (OR = 0.955, 95% CI [0.936, 0.975], $p < .001$): each additional year of estimated age was associated with a 4.5% reduction in the odds of achieving adequate knowledge. Equivalently, a 10-year age increase reduced the odds by approximately 37% ($OR^{10} \approx 0.63$). Female sex showed a non-significant trend toward higher odds of adequate knowledge (OR = 1.52, $p = .130$).

The convergence of the OLS and logistic models in identifying age as the principal independent predictor, whether knowledge is modeled as a continuous score or as a binary adequacy threshold, strengthens confidence in this finding and satisfies the reviewer's recommendation for multivariable adjustment. Together, these results indicate that older age is the most actionable target for pharmacovigilance education, independent of sex and education.

**Table S4**

*Age- and Sex-Adjusted Odds Ratios for Correct Response on Individual Knowledge Items (n = 332)*

| | Sex (female) | | Age | | |
|---|---|---|---|---|---|
| **Knowledge item** | **OR** | **95% CI** | **OR** | **95% CI** | ***p* (age)** |
| NSAIDs cause GI damage | 1.840* | [1.065, 3.177] | 0.986 | [0.966, 1.005] | .153 |
| NSAIDs safe for kidneys (F) | 0.834 | [0.491, 1.414] | 0.963*** | [0.945, 0.982] | <.001 |
| Paracetamol non-toxic (F) | 1.542 | [0.899, 2.645] | 0.963*** | [0.944, 0.982] | <.001 |
| Paracetamol liver damage | 1.469 | [0.846, 2.550] | 0.977* | [0.957, 0.996] | .021 |
| Paracetamol allergy | 1.815* | [1.070, 3.077] | 0.977* | [0.958, 0.995] | .014 |

*Note.* Each row represents a separate binary logistic regression with the item (correct = 1, incorrect/unsure = 0) as the dependent variable and sex (female = 1) and age (continuous) as simultaneous predictors. (F) = correct answer is False. *$p < .05$. ***$p < .001$.

Item-level logistic regressions adjusted for both age and sex revealed a consistent and informative pattern. Age was a statistically significant independent predictor for four of five items: NSAID renal safety (OR = 0.963, $p < .001$), paracetamol toxicity recognition (OR = 0.963, $p < .001$), paracetamol liver damage (OR = 0.977, $p = .021$), and paracetamol allergy (OR = 0.977, $p = .014$). For the two items with the strongest age effects (renal safety and toxicity), each decade of age was associated with approximately 31% lower odds of a correct response ($OR^{10} \approx 0.69$).

Female sex, after adjusting for age, was independently associated with higher correct response rates on two items: NSAID GI damage (OR = 1.84, $p = .029$) and paracetamol allergic reactions (OR = 1.82, $p = .027$). The bivariate gender differences previously observed on the remaining three items were attenuated to non-significance after age adjustment, indicating that the crude gender effect was partially confounded by the younger age distribution of females in this sample (female *M* age = 30.2 vs. male *M* age = 43.1; $p < .001$).

This is a critical interpretive finding that the unadjusted analyses could not reveal. The bivariate results (Table 6 in the main manuscript) suggested a broad gender knowledge gap across most items. The adjusted analyses show that much of this apparent gender effect was driven by age confounding: because females in this sample were substantially younger and younger age is the dominant predictor of higher knowledge, the unadjusted female advantage was inflated. After removing the age confound, female sex independently predicted better knowledge on only two items, both involving adverse reaction awareness

(GI damage and allergic reactions). This suggests that the residual female advantage may reflect sex-specific health information exposure, such as through reproductive healthcare encounters or greater engagement with medication safety information, rather than a generalized knowledge superiority.
